# Sero-molecular survey on *Toxoplasma gondii* infection among drug addicted and non-addicted individuals: a case–control study

**DOI:** 10.1186/s12879-021-06979-8

**Published:** 2022-01-04

**Authors:** Majid Sharifzadeh, Hassan Rezanezhad, Kavous Solhjoo, Zahra Kargar Jahromi, Enayatollah Shadmand, Saeed Shahabi, Ali Taghipour

**Affiliations:** 1 Department of Medical Parasitology and Mycology, School of Medicine, Jahrom University of Medical Sciences, Jahrom, Iran; 2 Zoonoses Research Center, Jahrom University of Medical Sciences, Jahrom, Iran; 3 Department of Medical Entomology and Vector Control, School of Health, Shiraz University of Medical Sciences, Shiraz, Iran

**Keywords:** Toxoplasmosis, Drug addicted individuals, Seroprevalence, Molecular prevalence

## Abstract

**Background:**

Up to now, epidemiological studies on the prevalence of *Toxoplasma gondii* infection among drug addicted individuals have been rarely performed. By designing an age and sex matched case–control study, we sought to determine the prevalence and associated factors with *T. gondii* infection in these population using serological and molecular techniques.

**Methods:**

One hundred and thirty-seven drug addicted individuals and 141 healthy subjects were enrolled in this study. Informed consent as well as a standard questionnaire were obtained from all subjects participating. Blood samples were collected from each participant and the serum was screened for anti-*Toxoplasma* antibodies (IgG and IgM). PCR assay was performed using the primer pair targeting the RE and GRA6 genes of *T. gondii*. Then, PCR products were sequenced to determine genotype.

**Results:**

The seroprevalence of *T. gondii* infection based on IgG titer was 34.3% in case and 9.9% in the control groups, revealing a statistically significant difference (OR = 4.37; 95% CI = 2.46–9.12; P = 0.001). After analyzing the variables studied through the questionnaire, age was the only significantly factor associated with the anti-*T. gondii* IgG antibody in case group. Considering PCR assays with RE genomic target, the prevalence of *T. gondii* infection was 5.1% in the case and 3.5% in control groups which the difference was no statistically significant (OR = 1.46; 95% CI = 0.45–4.73; P = 0.521). Subsequently, all sequenced samples were genotype #1 using the GRA6 genomic target.

**Conclusions:**

*T. gondii* exposure is relatively high among drug addicted individuals in Iran, and there is a need for health policymakers and researchers to establish enlightenment and prevention programs for these population at risk of infection.

**Supplementary Information:**

The online version contains supplementary material available at 10.1186/s12879-021-06979-8.

## Background

*Toxoplasma gondii* (*T. gondii*), a cosmopolitan intracellular protozoan belonging to the phylum Apicomplexa, is an opportunistic pathogen that infects humans and a wide range of warm-blooded animals, including livestock, birds, rodents, and aquatic mammals [1, 2]. According to published literatures, this zoonotic parasite affects approximately one-third of the world’s population, especially in communities with low socioeconomic level in many developing countries [3, 4]. The life cycle of *T. gondii* consists of the felids (definitive hosts) and warm-blooded vertebrates (intermediate hosts) [5], and pollution of environmental resources occurs only by the felids via shedding oocysts [6, 7].

Human toxoplasmosis occurs mostly via the intake of undercooked or raw meat products infected with tissue cysts [8], or by ingestion of soil, water, or vegetables infected with sporulated oocysts [9]. Moreover, *T. gondii* can be passed through transplacental transmission, blood transfusions, and organ transplantation [10–12]. *T. gondii* infection is usually asymptomatic in immunocompetent individuals, but the parasite may induce neurological and ocular disorders in fetuses and immunocompromised patients [13, 14]. Moreover, numerous studies have suggested that this protozoan plays a potential role in increasing the incidence of psychiatric diseases (e.g. schizophrenia, autism, and major depression), suicide, traffic accidents, and addiction [15–19].

Narcotic drugs are serious public health threat in many countries, notably in Asia (Iran, Afghanistan, Pakistan, and India) [20, 21]. Many research studies have shown that narcotic drug addiction can disrupt the immune system and provides suitable conditions for infectious diseases [22–24]. In this regard, narcotic drugs addiction and *T. gondii* infection are endemic in different parts of Iran. According to published meta-analysis studies, the seroprevalence of *T. gondii* in the general population [25], pregnant women [26], and immunocompetent individuals [27] has been reported as 39.3%, 41% and 50.01% in Iran, respectively. To the best of our knowledge, very few studies have been performed on the status of *T. gondii* infection in drug addicted individuals as a high-risk population [28, 29]. Therefore, the present case–control study was conducted to assess the prevalence, sociodemographic, and risk factors associated with this infection in drug addicted and non-addicted individuals using both serologic and molecular techniques.

## Methods

### Study population and sample collection

The current case–control study obtained ethical approval from the Ethics Committee of the Jahrom University of Medical Sciences, Jahrom, Iran (No. IR.JUMS.REC.1397.154). All sampling processes was done during April 2019 to December 2019 from drug addicted individuals referred to the laboratories of the medical centers of Lar city in Fars Province, Iran. Regarding the criteria, confirmed addiction test and consent for participating were considered as inclusion criteria, and presence of immunodeficiency diseases were considered as exclusion criteria. G*Power software was applied to calculate the sample size in this case–control study. We used prevalence reference of 7.7% (IgG) [28], at a confidence intervals (CI) of 95%, type I error-*α* of 0.05, and the power of the test (1−β) of 90% to estimate the sample size. The calculation outcome was 135 individuals in each group, although 137 confirmed drug addicted individuals and 141 non-addicted individuals were employed for more accuracy. The control group were people who had no history of addiction. For a more accurate assessment, the age and sex of the control group were matched with the case group. An informed consent form as well as a standard questionnaire were designed and prepared for all participants. Each participant was interviewed orally by the interviewer. This questionnaire included the sociodemographic variables, and risk factors related to *T. gondii* infection. Afterwards, approximately 10 cc of blood samples were gathered of each participant with and without EDTA. At first, a rapid HIV test was performed on samples from all participants, and if the result was negative, they entered the next stage. Then, the all samples were transported to the laboratory via the cold chain conditions. Blood samples were permitted to clot and centrifuged at 600×*g* for 10 min to extract the serum and buffy coats [30]. Finally, the separated serum (without EDTA) for serological assay and buffy coats (with EDTA) for PCR-based molecular assay were stored at − 20 °C until examination.

### Serological assay

All serum samples were surveyed for anti-*Toxoplasma* IgG and IgM antibodies through a commercially available enzyme-linked immunosorbent assay (ELISA) kits (Pishtazteb Co, Iran) according to the manufacturer's protocol. This ELISA kits had the 100% sensitivity and 99% specificity. Each run was accompanied by positive and negative controls. Anti-*T. gondii* IgG and IgM antibodies were estimated via dividing the sample optical density value by the cut-off calibrator value. Considering the kit protocol, when an IgG index or an IgM index was ≥ 1.1, we considered that sample to be positive for IgG or IgM.

### Molecular assay

The DNA was extracted from buffy coat samples through the CinnaPure-DNA extraction kit (CinnaGen Co., Tehran, Iran), as stated in the manufacturer’s protocol. The concentration and quality of the DNA were determined using the NanoDrop spectrophotometer. We used two genes RE and GRA6 for molecular assessment. DNA extracted from the *T. gondii* tachyzoites standard RH-strain and deionized sterile water were used for each run as positive and negative controls, respectively.

To detection of *T. gondii*, the PCR reaction was arranged in a 20 μL total reaction volume comprising 10 μL of Master mix, 5 μL of DNA, 3 μL deionized sterile water, and 1 μL of each primer TOXO4 (forward: 5′-CGCTGCAGGGAGGAAGACGAAAGTTG-3′) and TOXO5 (reverse: 5′-CGCTGCAGACACAGTGCATCTGGATT-3′) targeting the RE gene of *T. gondii* [31]. Thermal cycling profile was 94 °C for 5 min, followed by 35 cycles of 30 s at 94 °C, 30 s at 63 °C and 30 s at 72 °C with a final step at 72 °C for 10 min. The PCR amplification is characterized by the production of a 529 bp band for the RE gene [31].

To identify the genotypes of *T. gondii* isolates, nested PCR were conducted by a set of specific primers targeting a GRA6 gene of *T. gondii*. At the first step, PCR was performed using GRA6-F1 (forward: 5′-ATTTGTGTTTCCGAGCAGGT-3′) and GRA6-R1 (reverse: 5′-GCACCTTCGCTTGTGGTT-3′), which together amplify a 546-bp specific fragment [32]. Amplification was accomplished in final volume of 20 μL, comprising 10 μL of Master mix, 5 μL of DNA, 3 μL deionized sterile water, and 1 μL of each primer. The PCR amplification conditions were 94 °C for 5 min followed by 40 cycles of 94 °C for 30 s, 56 °C for 30 s, and 72 °C for 30 s, and final extension of 72 °C for 10 min [32]. In the next step, a second PCR was performed using the forward primer GRA6-F2 (5′-TTTCCGAGCAGGTGACCT-3′) and the reverse primer GRA6-R2 (5′-TCGCCGAAGAGTTGACATAG-3′) in order to amplify a 344-bp internal fragment used for genotype identification [32]. The second PCR reaction used 94 °C for 5 min, followed by 30 cycles of 94 °C for 30 s, 59 °C for 30 s, and 72 °C for 30 s, plus a final extension step at 72 °C for 10 min [32].

At the end, PCR products (both RE and GRA6 genes) were electrophoresed on a1.2% agarose gel with SYBR^®^ Safe stain and visualized using a UV Transilluminator.

### Sequencing

PCR products of positive samples were sequenced using ABI 3730 Analyzers (Bioneer, Korea) and the results were compared with sequences available in the GenBank database by BLAST software.

### Statistical analysis

To data the analysis, the Chi-square and Fisher’s exact tests were performed using SPSS 18 software (SPSS, Chicago, IL, USA). The possible associations were estimated through odds ratios (OR) and 95% CI after adjustments. A *P*-value of < 0.05 was considered statistically significant for differences.

## Results

A total of 278 participants were involved in this study. Among these, 137 individuals were addicted with narcotic drugs (78.1% male and 21.9% female) and 141 individuals were non-addicted (78% male and 22% female). Most addicts used opium (81.8%) and the main route of use was inhalation (48.9%). More sociodemographic characteristics and risk factors of *T. gondii* seroprevalence among addicted and non-addicted individuals are summarized in Table 1 and Additional file 1: Table S1, respectively. The seroprevalence of anti-*T. gondii* IgG antibody in drug addicted individuals 34.3% (47/137) was higher than in non-addicted individuals 9.9% (14/141), a statistically significant difference was observed (OR = 4.37; 95% CI = 2.46–9.12; P = 0.001). Moreover, although the seroprevalence of *T. gondii* infection based on IgM antibody titer was 1.5% (2/137) in case group, no titer was observed in the control group, revealing no statistically significant association (OR = 0.49; 95% CI = 0.43–0.55; P = 0.242). As shown in Table 1, there was only a statistically significant difference between age and anti-*T. gondii* IgG antibody in case group. Considering PCR assays with RE genomic target (Table 2), the prevalence of *T. gondii* infection was 5.1% (7/137) in case subjects and 3.5% (5/141) in the healthy control subjects, there was no statistically significant difference between case and control groups (OR = 1.46; 95% CI = 0.45–4.73; P = 0.521). Molecular prevalence of *T. gondii* according to sociodemographic characteristics and risk factors for addicted and non-addicted individuals are presented in Table 2 and Additional file 2: Table S2, respectively. Then, genotyping was performed by the GRA6 genomic target on 12 positive molecular samples of case and control groups. In this regard, only 4 samples (2 samples from each case and control group) were genotyped. All four samples were genotype #1, which were submitted with accession numbers MW311068, MW311069, MW311070, and MW311071 in the GenBank.

**Table 1:** Sociodemographic and risk factors of *Toxoplasma gondii* seroprevalence among drug addicted individuals referred to the laboratories of the medical centers of Lar city in Fars Province during April 2019 to December 2019

Characteristic		IgG				*p*-value	IgM				*p*-value
		Positive		Negative			Positive		Negative		
		Frequency	%	Frequency	%		Frequency	%	Frequency	%	
Gender	Male	35	32.7	72	67.3	0.457	1	99.1	0.391	1.99	0.391
	Female	12	40.0	18	60.0		1	96.7		7.96	
Married	No	9	29.0	22	71.0	0.482	0	100.	0.99	.100	0.99
	Yes	38	35.8	68	64.2		2	98.1		1.98	
Age (year)	30 >	1	7.7	12	92.3	0.039*	0	100	0.223	100	0.223
	31–40	15	26.3	42	73.7		0	100		100	
	41–50	17	47.2	19	52.8		2	94.4		4.94	
	51–60	9	47.4	10	52.6		0	100		100	
	60 <	5	41.7	7	58.3		0	100		100	
Education	Illiterate	1	20.0	4	80.0	0.491	0	100	0.314	100	0.314
	Primary school	18	43.9	23	56.1		2	95.1		1.95	
	Secondary school	15	27.3	40	72.7		0	100		100	
	Diploma	11	36.7	19	63.3		0	100		100	
	College	2	33.3	4	66.7		0	100		100	
Occupation	Self-employed	39	33.6	77	66.4	0.289	2	98.3	0.947	3.98	0.947
	Unemployed	1	33.3	2	66.7		0	100		100	
	Housewife	2	20.0	8	80.0		0	100		100	
	Employee	5	62.5	3	37.5		0	100		100	
Residence	Urban	30	34.1	58	65.9	0.943	0	100	0.056	100	0.056
	Rural	17	34.7	32	65.3		2	95.9		9.95	
Consumption of meat	Grilled	12	35.3	22	64.7	0.498	1	97.1	0.624	1.97	0.624
	Boiled	28	37.3	47	62.7		1	98.7		7.98	
	Grilled/boiled	7	25.0	21	75.0		0	100		100	
Type of water source	Well water	8	29.6	19	70.4	0.164	0	100	0.799	100	0.799
	Cistern	8	23.5	26	76.5		1	97.1		1.97	
	Treated pipe water	30	42.9	40	57.1		1	98.6		6.98	
	Mineral water	1	16.7	5	83.3		0	100		100	
Type of vegetable wash	Only water	39	35.5	71	64.5	0.568	2	98.2	0.99	98.2	0.99
	With disinfectant	8	29.6	19	70.4		0	100		100	
Contact with cat	No	28	33.3	56	66.7	0.763	1	98.8	0.99	98.8	0.99
	Yes	19	35.8	34	64.2		1	98.1		98.1	
Duration of consumption of narcotic drug	Less than a year	1	25.0	3	75.0	0.99	0	100		100	0.99
	More than a year	46	34.6	87	65.4		2	98.5		98.5	
The way of consumption of narcotic drugs	Inhalation	27	40.3	40	59.7	0.141	1	98.5	0.862	98.5	0.862
	Oral	9	37.5	15	62.5		0	100		100	
	Oral-inhalation	8	20.0	32	80.0		1	97.5		97.5	
	Injection- inhalation	3	50.0	3	50.0		0	100		100	
Type of the consumed narcotic drug	Opium	40	35.7	72	64.3	0.502	2	98.2	0.797	98.2	0.797
	Heroin	2	18.2	9	81.8		0	100		100	
	Crystal methamphetamine	5	35.7	9	64.3		0	100		100	

^*^Statistically significant difference (*P*-value of < 0.05)

**Table 2:** Sociodemographic and risk factors of drug addicted individuals and molecular-prevalence of *T*. *gondii* infection referred to the laboratories of the medical centers of Lar city in Fars Province during April 2019 to December 2019

Characteristic		PCR with RE genomic target				*P*-value
		Positive		Negative		
		Frequency	%	Frequency	%	
Gender	Male	5	4.7	102	95.3	0.661
	Female	2	6.7	28	93.3	
Married	No	2	6.5	29	93.5	0.700
	Yes	5	4.7	101	95.3	
Age (year)	30 >	0	0	13	100	0.658
	31–40	4	7.0	53	93.0	
	41–50	2	6.5	34	94.4	
	51–60	0	0	19	100	
	60 <	1	8.3	11	91.7	
Education	Illiterate	0	0	5	100	0.876
	Primary school	3	7.3	38	92.7	
	Secondary school	3	5.5	52	94.5	
	Diploma	1	3.3	29	96.7	
	College	0	0	6	100	
Occupation	Self-employed	7	6.0	109	94.0	0.721
	Unemployed	0	0	3	100	
	Housewife	0	0	10	100	
	Employee	0	0	8	100	
Residence	Urban	4	4.5	84	95.5	0.700
	Rural	3	6.1	46	93.9	
Consumption of meat	Grilled	4	11.8	30	88.2	0.125
	Boiled	2	2.7	73	97.3	
	Grilled/boiled	1	3.6	27	96.4	
Type of water source	Well water	2	7.4	2	92.6	
	Cistern	1	2.9	33	97.1	
	Treated pipe water	4	5.7	66	94.3	0.801
	Mineral water	0	0	6	100	
Type of vegetable wash	Only water	7	6.4	103	93.6	0.178
	With disinfectant	0	0	27	100	
Contact with cat	No	5	6.0	79	94.0	0.706
	Yes	2	3.8	51	96.2	
Duration of consumption of narcotic drug	Less than a year	0	0	4	100	0.99
	More than a year	7	5.3	126	94.7	
The way of consumption of narcotic drugs	Inhalation	3	4.5	64	95.5	0.827
	Oral	1	4.2	23	95.8	
	Oral-inhalation	3	7.5	37	92.5	
	Injection-inhalation	0	0	6	100	
Type of the consumed narcotic drug	Opium	7	6.3	105	93.8	0.439
	Heroin	0	0	11	100	
	Crystal methamphetamine	0	0	14	100	

## Discussion

The prevalence of *T. gondii* infection and associated factors in drug addicted individuals are largely unknown. Consequently, the present case–control study attempted to determine the sero-molecular prevalence of *T. gondii* and the prevalence association with the sociodemographic characteristics and risk factors in addicted and non-addicted individuals in Lar, Iran. As the results of this study showed, exposure to *T. gondii* was higher in drug addicted individuals than in non-addicted individuals, which was significant for IgG. Addicted individuals may be more exposed to *T. gondii* infection due to poverty, poor health and nutritional condition. In Iran, one study has been performed on the relationship between *T. gondii* infection and addicted women [29]; the methodology and results were different from our study. In their study, there was no significant relationship between the seroprevalence of anti-*T. gondii* IgG antibody in the case and control groups as well as molecular assay were not performed. However, this difference should be interpreted with caution because various factors such as methodology (study design), social, economic, geographical and demographic can play a intervening role in the difference prevalences [3]. In 2015, a meta-analysis of the relationship between *T. gondii* infection and addiction was performed with only four included articles [19]. The results of meta-analysis showed that there is a significant OR between the increased prevalence of *T. gondii* infection and addiction (OR = 1.91; 95% CI = 1.49–2.44; P = 0.00001). However, in future research, it is suggested that more epidemiological studies be conducted on the prevalence of *T. gondii* in these high-risk group to gain in-depth insights into these relationships.

As shown in Tables and Additional file 1: Table S1 and Additional file 2: Table S2, we collected a wide range of sociodemographic characteristics and risk factors in both case and control groups, considering both serological and molecular techniques. Although only age was significantly associated with the anti-*T. gondii* IgG antibody in case group, other variables should not be ignored. In this regard, cats as the definitive hosts of *T. gondii*, play a key role in the transmission of sporulated oocysts to humans as well as polluting the environment [6]. A global meta-analysis study on contamination of soil with *T. gondii* oocysts was performed in different public places of the world [7]; in this regard, the estimated prevalences in Europe, South America, Asia and North America were 23% (95% CI 4–65), 22% (95% CI 18–26), 15% (95% CI 0.06–33) and 8% (95% CI 0.00–97), respectively. This indicates that most of the different regions in the world, especially public parks, are contaminated with *T. gondii* oocysts and the chance of acquiring the infection are high, especially in drug addicted individuals who often present in such environments. Due to the transmission of *T. gondii* through the blood, injecting drug users are more likely than other addicts to become infected due to the frequent use of contaminated syringes. However, injecting drug users participating in the present study had the lowest sample size compared to other addicts. Hence, a detailed assessment of the prevalence of *T. gondii* with higher sample size in injecting drug users is suggested.

In the present study, 1.5% of drug addicted individuals had IgM antibodies against *T. gondii*. It should be noted that the absence of IgM in people with anti-*T. gondii* IgG antibodies shows a chronic infection, but its presence does not necessarily indicate an acute infection [33]. Moreover, PCR results indicated that a relatively high number of cases (5.1%) and controls (3.5%) harbor circulating DNA of *T. gondii*. One of the most important reasons for the difference in prevalence between serological and molecular methods is the difference in sensitivity and specificity of these techniques [34]. Given that good PCR sensitivity is important for the detection of circulating DNA in people with chronic toxoplasmosis [34]. In the present study, all sequenced samples were from genotype #1. Due to the fact that genotype #1 has more virulence capability than other genotypes (#2 and #3) and is often isolated in patients with immunodeficiency [32], it is suggested that more molecular epidemiology be done on addicts to better understand the distribution status of genotypes.

## Conclusions

Our findings showed a high sero-molecular prevalence of *T. gondii* in addicted individuals in Iran. Such studies need to be done on a larger sample in other parts of the world and there is a need for health policymakers and researchers to establish enlightenment and prevention programs for these population at risk of infection.

## Supplementary Information

**Additional file 1: Table S1**. Sociodemographic and risk factors of *Toxoplasma gondii *seroprevalence among non-addicted individuals referred to the laboratories of the medical centers of Lar city in Fars Province during April 2019 to December 2019.

**Additional file 2: Table S2**. Sociodemographic and risk factors of non-addicted individuals and molecular-prevalence of *T*. *gondii *infection referred to the laboratories of the medical centers of Lar city in Fars Province during April 2019 to December 2019.

## Data Availability

All data is available in the text of the manuscript, Tables, and Additional files.
