# Supplementary material for: Sero-molecular survey on Toxoplasma gondii infection among drug addicted and non-addicted individuals: a case–control study
Source: BMC Infect Dis. 2022 Jan 4;22:19. doi: 10.1186/s12879-021-06979-8 (PMC8725485; doi:10.1186/s12879-021-06979-8)
Supplement: Supplementary file 1 — Additional file 1: Table S1. Sociodemographic and risk factors of Toxoplasma gondii seroprevalence among non-addicted individuals referred to the laboratories of the medical centers of Lar city in Fars Province during April 2019 to December 2019. [file 12879_2021_6979_MOESM1_ESM.docx]

| Supplementary Table 1. Sociodemographic and risk factors of *Toxoplasma gondii* seroprevalence among non-addicted individuals referred to the laboratories of the medical centers of Lar city in Fars Province during April 2019 to December 2019. | | | | | | |
| --- | --- | --- | --- | --- | --- | --- |
| *P*-value | IgG | | | | Characteristic | |
|  | **Negative** | | **Positive** | |  |  |
|  | **%** | Frequency | % | Frequency |  |  |
| 464.0 | 1.89 | 98 | 9.10 | 12 | Male | Gender |
|  | 5.93 | 29 | 5.6 | 2 | Female |  |
| 0.847 | 7.91 | 11 | 3.8 | 1 | No | Married |
|  | 9.89 | 116 | 1.10 | 13 | Yes |  |
| 076.0 | 3.92 | 12 | 7.7 | 1 | 30> | Age (year) |
|  | 5.92 | 49 | 5.7 | 4 | 40-31 |  |
|  | 4.78 | 29 | .621 | 8 | 50-41 |  |
|  | 3.96 | 26 | 7.3 | 1 | 60-51 |  |
|  | 100 | 11 | 0 | 0 | 60< |  |
| 981.0 | 0 | 0 | 0 | 0 | Illiterate | Education |
|  | 5.87 | 7 | 5.12 | 1 | Primary school |  |
|  | 5.88 | 23 | 5.11 | 3 | Secondary school |  |
|  | 5.90 | 57 | 5.9 | 6 | Diploma |  |
|  | 9.90 | 40 | 1.9 | 4 | College |  |
| 575.0 | 8.88 | 87 | 2.11 | 11 | Self-employed | Occupation |
|  | 0.80 | 4 | 0.20 | 1 | Unemployed |  |
|  | 100 | 8 | 0 | 0 | Housewife |  |
|  | 3.93 | 28 | 7.6 | 2 | Employee |  |
| 0.390 | 6.91 | 87 | 4.8 | 8 | Urban | Residence |
|  | 0.87 | 40 | 0.13 | 6 | Rural |  |
| 674.0 | 0.92 | 23 | 0.8 | 2 | Grilled | Consumption of meat |
|  | 3.87 | 48 | 7.12 | 7 | Boiled |  |
|  | 8.91 | 56 | 2.8 | 5 | Grilled/boiled |  |
| 390.0 | 0.90 | 18 | 0.10 | 2 | Well water | Type of water source |
|  | 6.94 | 35 | 4.5 | 2 | Cistern |  |
|  | 5.86 | 64 | 5.13 | 10 | Treated pipe water |  |
|  | 100 | 10 | 0 | 0 | Mineral water |  |
| 761.0 | 4.90 | 104 | 6.9 | 11 | Only water | Type of vegetable wash |
|  | 88.5 | 23 | 5.11 | 3 | With disinfectant |  |
| 787.0 | 3.90 | 112 | 7.9 | 12 | No | Contact with cat |
|  | 2.88 | 15 | 8.11 | 2 | Yes |  |
