# Supplementary material for: Sero-molecular survey on Toxoplasma gondii infection among drug addicted and non-addicted individuals: a case–control study
Source: BMC Infect Dis. 2022 Jan 4;22:19. doi: 10.1186/s12879-021-06979-8 (PMC8725485; doi:10.1186/s12879-021-06979-8)
Supplement: Supplementary file 2 — Additional file 2: Table S2. Sociodemographic and risk factors of non-addicted individuals and molecular-prevalence of T. gondii infection referred to the laboratories of the medical centers of Lar city in Fars Province during April 2019 to December 2019. [file 12879_2021_6979_MOESM2_ESM.docx]

| Supplementary Table 2. Sociodemographic and risk factors of non-addicted individuals and molecular-prevalence of *T*. *gondii* infection referred to the laboratories of the medical centers of Lar city in Fars Province during April 2019 to December 2019. | | | | | | |
| --- | --- | --- | --- | --- | --- | --- |
| *P*-value | PCR with RE genomic target | | | | Characteristic | |
|  | **Negative** | | **Positive** | |  |  |
|  | **%** | Frequency | % | Frequency |  |  |
| 0.303 | 3.97 | 107 | 7.2 | 3 | Male | Gender |
|  | 5.93 | 29 | 5.6 | 2 | Female |  |
| 0.99 | 100 | 12 | 0 | 0 | No | Married |
|  | 1.96 | 124 | 9.3 | 5 | Yes |  |
| 799.0 | 100 | 13 | 0 | 0 | 30> | Age (year) |
|  | 3.94 | 50 | 7.5 | 3 | 40-31 |  |
|  | 3.97 | 36 | 7.2 | 1 | 50-41 |  |
|  | 3.96 | 26 | 7.3 | 1 | 60-51 |  |
|  | 100 | 11 | 0 | 0 | 60< |  |
| 854.0 | 100 | 0 | 0 | 0 | Illiterate | Education |
|  | 100 | 8 | 0 | 0 | Primary school |  |
|  | 2.96 | 25 | 8.3 | 1 | Secondary school |  |
|  | 2.95 | 60 | 8.4 | 3 | Diploma |  |
|  | 7.97 | 43 | 3.2 | 1 | College |  |
| 0.517 | 9.94 | 93 | 1.5 | 5 | Self-employed | Occupation |
|  | 100 | 5 | 0 | 0 | Unemployed |  |
|  | 100 | 8 | 0 | 0 | Housewife |  |
|  | 100 | 30 | 0 | 0 | Employee |  |
| 661.0 | 8.96 | 92 | 2.3 | 3 | Urban | Residence |
|  | 7.95 | 44 | 3.4 | 2 | Rural |  |
| 0.534 | 100 | 25 | 0 | 0 | Grilled | Consumption of meat |
|  | 4.96 | 53 | 6.3 | 2 | Boiled |  |
|  | 1.95 | 58 | 9.4 | 3 | Grilled/boiled |  |
| 675.0 | 100 | 20 | 0 | 0 | Well water | Type of water source |
|  | 6.94 | 35 | 4.5 | 2 | Cistern |  |
|  | 9.95 | 71 | 1.4 | 3 | Treated pipe water |  |
|  | 100 | 10 | 0 | 0 | Mineral water |  |
| 229.0 | 4.97 | 112 | 6.2 | 3 | Only water | Type of vegetable wash |
|  | 3.92 | 24 | 7.7 | 2 | With disinfectant |  |
| 0.99 | 0.96 | 119 | 0.4 | 5 | No | Contact with cat |
|  | 100 | 17 | 0 | 0 | Yes |  |
